# Enhancing the Gastrointestinal Stability of Curcumin by Using Sodium Alginate-Based Nanoemulsions Containing Natural Emulsifiers

**DOI:** 10.3390/ijms24010498

**Published:** 2022-12-28

**Authors:** Júlia Teixé-Roig, Gemma Oms-Oliu, Isabel Odriozola-Serrano, Olga Martín-Belloso

**Affiliations:** Department of Food Technology, University of Lleida-Agrotecnio CERCA Center, 25198 Lleida, Spain

**Keywords:** curcumin, nanoemulsions, bioaccessibility, stability, sodium alginate

## Abstract

Curcumin presents interesting biological activities but low chemical stability, so it has been incorporated into different emulsion-based systems in order to increase its bioaccessibility. Many strategies are being investigated to increase the stability of these systems. Among them, the use of polysaccharides has been seen to highly improve the emulsion stability but also to modulate their digestibility and the release of the encapsulated compounds. However, the effect of these polysaccharides on nanoemulsions depends on the presence of other components. Then, this work aimed to study the effect of alginate addition at different concentrations (0–1.5%) on the gastrointestinal fate and stability of curcumin-loaded nanoemulsions formulated using soybean lecithin or whey protein as emulsifiers. Results showed that, in the absence of polysaccharides, whey protein was more effective than lecithin in preventing curcumin degradation during digestion and its use also provided greater lipid digestibility and higher curcumin bioaccessibility. The addition of alginate, especially at ≥1%, greatly prevented curcumin degradation during digestion up to 23% and improved the stability of nanoemulsions over time. However, it reduced lipid digestibility and curcumin bioaccessibility. Our results provide relevant information on the use of alginate on different emulsifier-based nanoemulsions to act as carriers of curcumin.

## 1. Introduction

Curcumin, chemically known as diferuloylmethane, is a low molecular weight (368.37 g·mol^−1^) polyphenol found in the rhizome of the *Curcuma longa* plant (turmeric). In its structure, it presents two aryl rings that contain *ortho*-methoxy phenolic groups symmetrically linked to a β-diketone moiety [1,2]. This compound has been shown to target multiple signalling molecules and presents antioxidant, anti-inflammatory, antibacterial and anticarcinogenic properties [3,4,5]. However, its incorporation in food matrices is difficult due to its poor water solubility and low chemical stability [6]. Furthermore, curcumin is highly unstable at the physiological pH [7], so it can be easily degraded in the intestinal tract. Oil-in-water emulsion-based systems appear to be feasible curcumin carriers due to the lipophilic nature of the compound. Indeed, many authors have studied the incorporation of curcumin into different encapsulation systems [6,8,9,10,11,12]. Among them, nanoemulsions are interesting options since they present higher physical stability against gravitational processes compared to conventional emulsions and enhanced functionality due to the increased surface area of droplets [13]. These systems have been seen to prevent curcumin degradation over time and increase its stability during gastrointestinal digestion, as well as enhance its bioaccessibility [6,14]. However, recent studies have highlighted that the nature of the emulsifier, the type and the amount of carrier oil and emulsifier–curcumin interactions are key factors that influence curcumin delivery in nanoemulsions [15,16].

Emulsifiers are needed to obtain stable nanoemulsions and can have a significant impact on their properties and digestibility, as well as on the bioaccessibility of bioactive compounds [2,16,17]. Among the possible emulsifiers to be used, naturals seem to be more suitable than synthetics because of general public concern about environmental issues and the demand for label-friendly food products. In this regard, soybean lecithin (SBL) and whey protein isolate (WPI) are natural emulsifiers with different chemical compositions previously used in the food industry. Lecithin is a lipid-based emulsifier that can be obtained from natural sources like soybean, sunflower or egg yolk and is able to stabilize emulsions forming a multilamellar shell around the droplets, acting as a mechanical barrier [18]. This emulsifier has been shown to provide good electrostatic stabilization due to its strong negative electrical charge in a wide range of pH [19,20,21]. Moreover, natural lecithin is biodegradable and among the safest emulsifying agents, being the incidence of allergic reactions to lecithin is very rare [18,22]. On the other hand, WPI is a protein-based emulsifier that consists of β-lactoglobulin and α-lactalbumin together with other minor proteins such as bovine serum albumin, lactoferrin and immunoglobulins [23]. This emulsifier can form a dense layer at the surface of the droplets, providing stability to emulsion-based systems for flocculation and coalescence [23,24]. Moreover, this emulsifier has been used to formulate nanoemulsions containing bioactive compounds such as β-carotene, shown to be highly effective in the prevention of compound degradation and providing high stability over time [25,26,27].

Polysaccharides of different natures have been used in the formulation of emulsion-based systems. For instance, they have been used as solid particle stabilizers in Pickering emulsions, showing to be promising ingredients to replace synthetic ingredients [10,11,28]. In addition, the incorporation of polysaccharides in other systems, such as nanoemulsions, has been seen to increase their physical stability by reducing the movement of droplets [29,30,31]. According to these studies, the addition of polysaccharides seems to be an interesting strategy for producing systems with enhanced stability. However, from a nutritional point of view, the digestibility of emulsion-based delivery systems can be affected by the type and concentration of polysaccharides added. According to previous literature, the effect of the polysaccharides depends on the emulsifier nature used to formulate the emulsions. Indeed, 0.2% fucoidan has been shown to increase lipid digestibility in caseinate emulsions but had no effect on Tween 20 or lecithin emulsions [32]. In the same way, other authors have observed an increased lipid digestibility by adding pectin in lecithin emulsions but a reduced digestibility when it was added to Tween 20 emulsions [33]. Based on these findings, the interactions that take place on the interface between the emulsifier and the polysaccharide can influence the digestibility of nanoemulsions. Previous authors have reported that the presence of polysaccharides, such as mandarin fibre or chitosan, in nanoemulsions can have an impact on the bioaccessibility of β-carotene or vitamin D, although controversial results were obtained [34,35].

Among the polysaccharides, the interest in the use of sodium alginate to encapsulate bioactive compounds has increased in the past two decades due to its biocompatibility, biodegradability and environmental sensitivity properties [36]. Moreover, this polysaccharide has been seen to be extremely effective in enhancing the stability of nanoemulsions [30,37]. However, there is a lack of knowledge on how the incorporation of sodium alginate in oil-in-water nanoemulsions may affect their digestibility and the bioaccessibility of the lipophilic encapsulated compound. Thus, the aim of the study was to investigate the effect of sodium alginate addition on the physical properties, stability and the digestibility of curcumin-loaded nanoemulsions formulated using different natural emulsifiers (soybean lecithin or whey protein isolate) and focus on their gastrointestinal stability and bioaccessibility of curcumin.

## 2. Results and Discussion

### 2.1. Initial Systems

#### 2.1.1. Particle Size and Distribution

**Figure 1:**
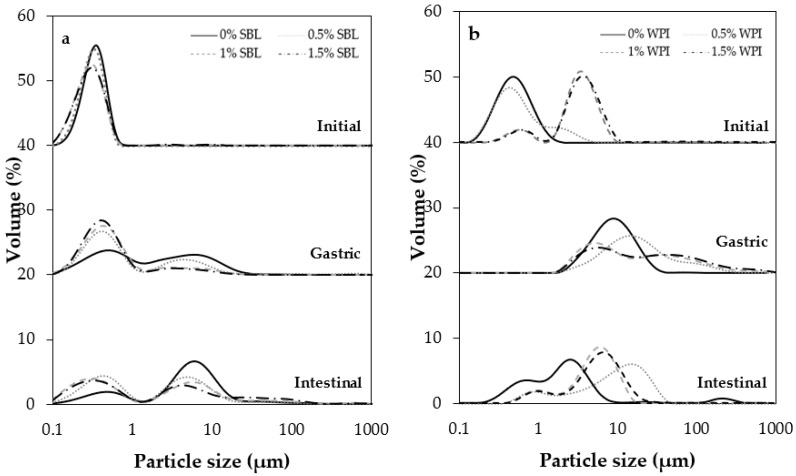
Particle size distribution of curcumin-loaded nanoemulsions stabilized with (**a**) soybean lecithin (SBL) or (**b**) whey protein isolate nanoemulsions (WPI) and different sodium alginate concentrations (0, 0.5, 1, and 1.5%) at different phases of in vitro digestion.

After the microfluidization process, SBL nanoemulsion without sodium alginate exhibited a smaller particle size (343.8 ± 9.7 nm) with a less polydisperse distribution than WPI nanoemulsion without the polysaccharide (462.4 ± 14.7 nm) (Table 1 and Figure 1). In addition, SBL reduced the interfacial tension (up to 14.66 mN/m) to a higher extent than WPI (up to 20.52 mN/m). The same trend was previously observed by other authors [38] and indicates that the higher surface activity of SBL may have facilitated the formation of small droplets rather than WPI.

**Figure 2:**
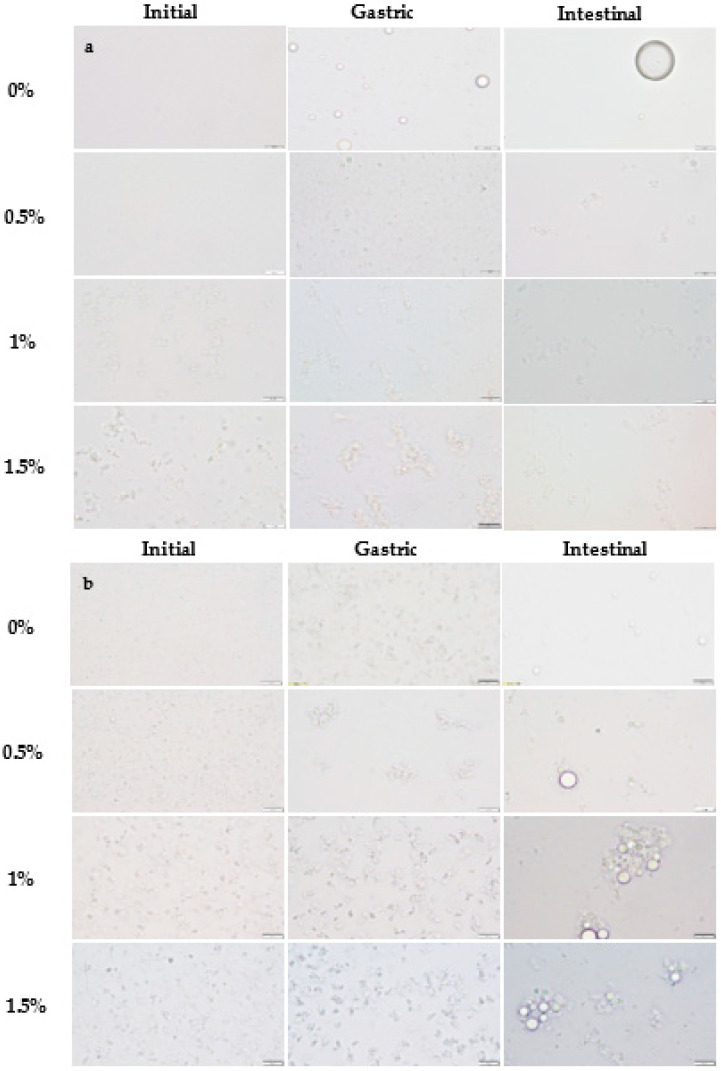
Microscope images of curcumin-loaded nanoemulsions stabilized with (**a**) soybean lecithin (SBL) or (**b**) whey protein isolate (WPI) and different sodium alginate concentrations (0, 0.5, 1 and 1.5%) at different phases of in vitro digestion. Scale bars were 10 µm long.

The addition of sodium alginate at any concentration significantly increased the particle size of WPI nanoemulsions due to the aggregation of droplets (Figure 2); the most noteworthy changes were observed when using 1% and 1.5% sodium alginate concentrations (Table 1). The particle size distribution changed from monomodal to bimodal when the polysaccharide was added to the WPI nanoemulsion, irrespective of the concentration used, leading to an increased polydispersity index. Conversely, sodium alginate addition had little effect on SBL nanoemulsions (Figure 1 and Table 1). As observed in the microscope images, all the obtained nanoemulsions exhibited flocculation when sodium alginate was added, regardless of the type of emulsifier and sodium alginate concentration (Figure 2). However, in SBL nanoemulsions, the mean particle size was not affected by the flocculation of droplets because aggregates were disrupted during the particle size measurements, as previously observed by other authors [32]. The aggregation of droplets observed in SBL and WPI nanoemulsions containing sodium alginate may be a consequence of non-adsorbed polymer molecules that were present in the aqueous phase surrounding the lipid droplets [39,40].

#### 2.1.2. ζ-Potential

Among non-added polysaccharide nanoemulsions formulated with SBL showed a more negative ζ-potential than WPI (Table 1). In SBL nanoemulsions, the negative charges conferred by the phosphate groups of phospholipids that were mostly negatively charged, except phosphatidylethanolamine and phosphatidylcholine, which were zwitterionic and neutral molecules at the pH of the nanoemulsions [12]. WPI presented a negative charge at the neutral pH of the nanoemulsions since the carboxyl groups were negatively charged (−COO^−^) and the amino groups were neutral (−NH_2_) at a pH above the isoelectric point of the protein (≈5) [41,42].

When adding sodium alginate, ζ-potential values of SBL and WPI nanoemulsions became more negative (Table 1). In both emulsifier-type nanoemulsions, the higher the polysaccharide concentration, the more negative the electrical charge was, up to values of −77.8 ± 4.3 mV and −57.7 ± 1.4 mV in SBL and WPI nanoemulsion with 1.5% polysaccharide, respectively. Sodium alginate has an anionic character due to the carboxylate and hydroxyl groups present on its molecule, so when it is adsorbed at the interface, it confers a negative charge [12]. Indeed, flocculation observed in the microscope images (Figure 2) proved that a layer of sodium alginate was surrounding the droplets. This phenomenon may be a consequence of non-adsorbed sodium alginate molecules forming a network structure surrounding the oil droplets.

#### 2.1.3. Encapsulation Efficiency

When sodium alginate was not present in the formulations, SBL nanoemulsion showed a slightly higher encapsulation efficiency (≈97%) than WPI nanoemulsion (≈95%) (Table 1). The phenolic hydroxyl group of curcumin can form H-bonds with the phosphate ion of lecithin so that the curcumin is surrounded by the two long aliphatic chains of lecithin, which may be favouring the retention of the compound within the droplets [12,43]. In contrast, WPI presented a less negative electrical charge than SBL, which indicates that it probably presented fewer hydroxyl groups that can be bound with curcumin. Moreover, since SBL presents a lower molecular weight and reduces more efficiently interfacial tension than WPI, the retention of curcumin would be higher in SBL nanoemulsion due to the more compact structure in the interface.

The addition of sodium alginate did not affect the encapsulation efficiency of the nanoemulsions regardless of the surfactant used. In fact, in previous studies, no significant differences were detected when increasing the concentration of sodium alginate, as in the case of gelatine-sodium alginate nanocapsules [44]. 

#### 2.1.4. Viscosity

Values of viscosity in nanoemulsions without sodium alginate were low and similar using SBL or WPI, both around 1.5 mPa·s (Table 1). The addition of sodium alginate noticeably increased the viscosity of nanoemulsions at any concentration, as expected. The higher the polysaccharide concentration, the higher the viscosity of the nanoemulsions was, up to a maximum of ≈118 mPa·s and ≈111 mPa·s in WPI and SBL nanoemulsions, respectively, containing 1.5% polysaccharide. In general, polysaccharides such as sodium alginate are used as thickening agents because they can increase the viscosity of the aqueous phase, hindering the movement of the droplets and, thereby, increasing the emulsion stability [45]. At intermediate polysaccharide concentrations (0.5–1%), SBL nanoemulsions presented a significantly lower viscosity than those formulated with WPI (Table 1). This fact may be attributed to the higher hydrodynamic diameter that WPI nanoemulsions presented in comparison to those with SBL (Table 1 and Figure 2), resulting in a higher viscosity [46]. Nevertheless, this difference was not observed when using the highest polysaccharide concentration (1.5%). In fact, in previous studies, similar viscosity values were observed when adding sodium alginate in nanoemulsions containing other emulsifiers such as Tween 20 [30]. This indicates that the viscosity of sodium-alginate-added nanoemulsions is governed by the concentration of the polysaccharide used, while the nature of the emulsifier used has little impact on the system viscosity.

#### 2.1.5. Stability

**Figure 3:**
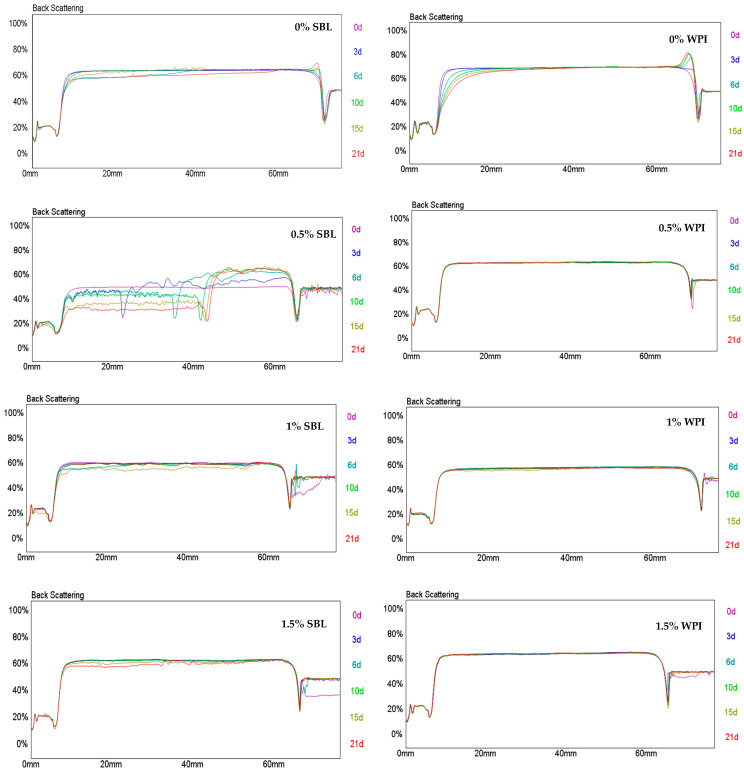
Variations on the backscattering profile of curcumin-loaded nanoemulsions stabilized with soybean lecithin (SBL) or whey protein isolate (WPI) and different sodium alginate concentrations (0, 0.5, 1 and 1.5%) during 21 days at 4 °C.

Both SBL and WPI nanoemulsions showed an increase in backscattering in the upper part of the test tube and a decrease in the lower part over time (Figure 3). This indicates that the droplets have migrated to the top, promoting creaming. However, it should be noted that this phenomenon was observed earlier and to a greater extent in WPI nanoemulsion (from day 3) than in SBL nanoemulsion (from day 6). Creaming is a common destabilisation phenomenon observed in nanoemulsions and can be the result of flocculation or coalescence [12,47]. In our study, WPI nanoemulsion presented a less negative charge than that with SBL (Table 1), so the repulsion between droplets may be lower in the nanoemulsion formulated with WPI rather than with SBL. Therefore, in WPI, nanoemulsion droplets may be more prone to aggregation and, consequently, to migrate to the top producing creaming.

The addition of sodium alginate increased the stability of WPI nanoemulsion over time, regardless of the concentration used. In this sense, although flocculation was observed in these nanoemulsions (Figure 2B), their high viscosity may have decreased the movement of droplets, preventing the creaming [48]. In contrast, the addition of polysaccharides enhanced the stability of SBL nanoemulsion when used at concentrations of ≥1% (Figure 3). As mentioned in the previous section, the viscosity of SBL nanoemulsions containing 0.5% sodium alginate was lower than that with WPI and the same polysaccharide concentration (Table 1). Therefore, it seems that the viscosity of SBL nanoemulsion containing 0.5% polysaccharide was not high enough to prevent the movement of the droplets.

### 2.2. Gastrointestinal In Vitro Digestion

#### 2.2.1. Physicochemical Changes during In Vitro Digestion

##### Gastric Phase

**Figure 4:**
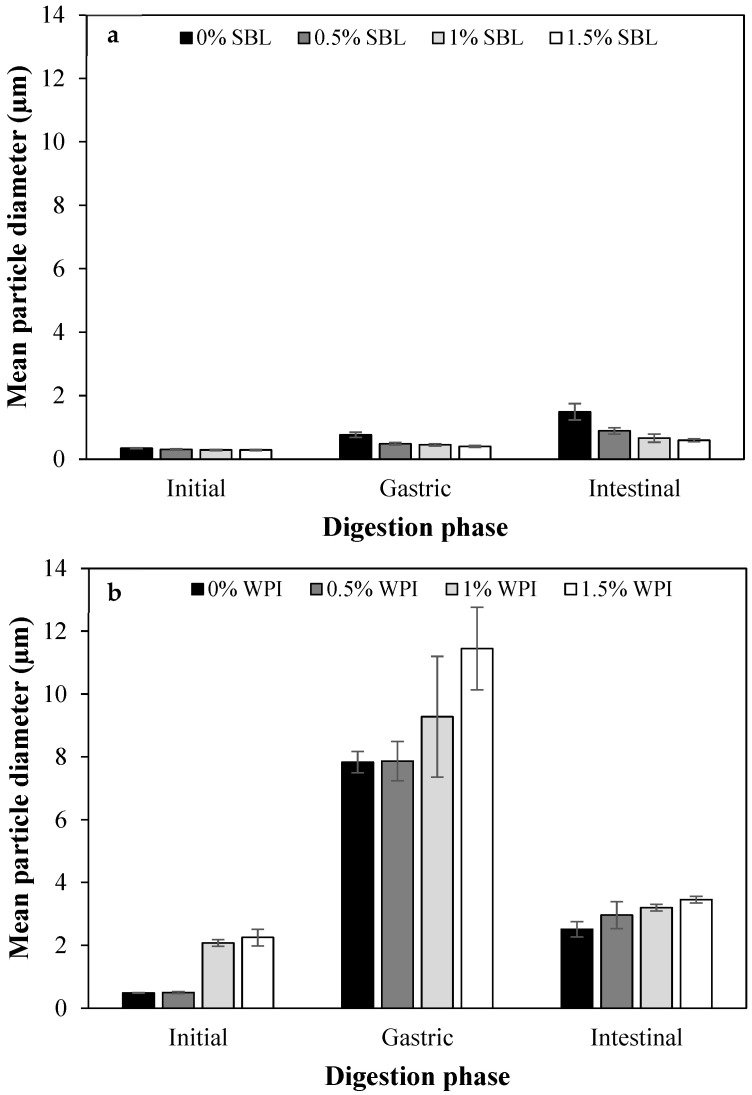
Particle size (µm) of curcumin-loaded nanoemulsions stabilized with (**a**) soybean lecithin (SBL) or (**b**) whey protein isolate (WPI) and different sodium alginate concentrations (0, 0.5, 1 and 1.5%) at different phases of in vitro digestion.

**Figure 5:**
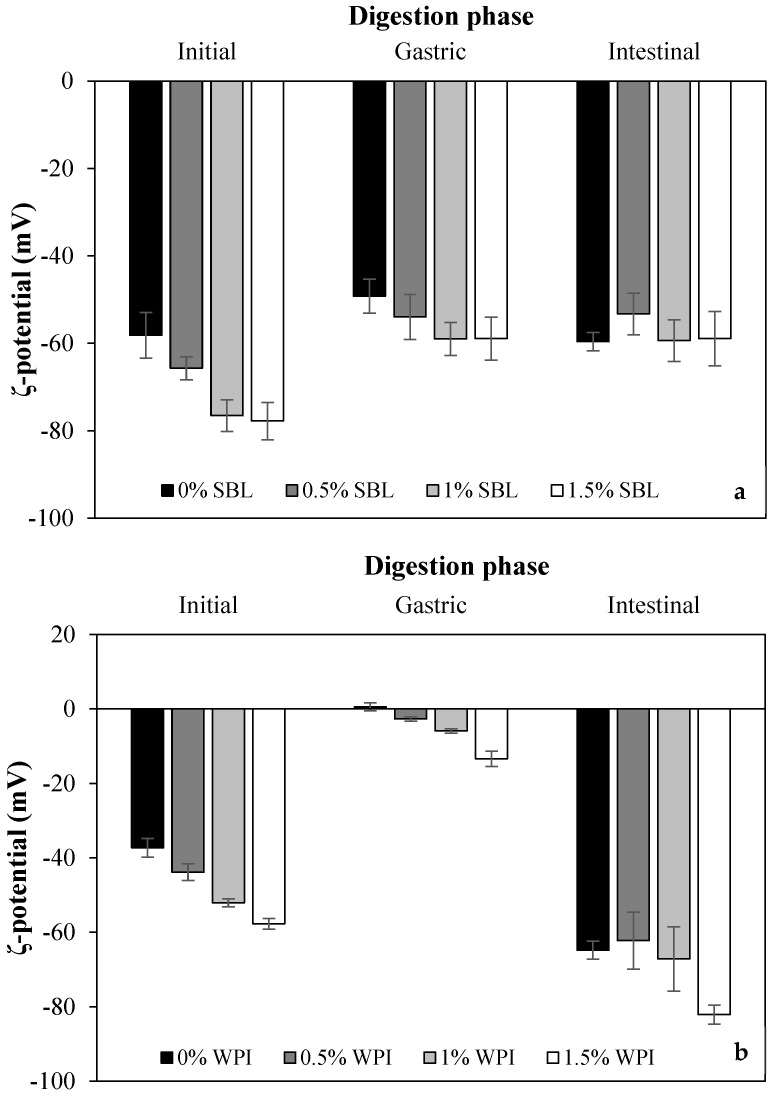
ζ-potential of curcumin-loaded nanoemulsions stabilized with (**a**) soybean lecithin (SBL) or (**b**) whey protein isolate (WPI) and different sodium alginate concentrations (0, 0.5, 1 and 1.5%) at different phases of in vitro digestion.

Among non-added polysaccharide nanoemulsions, those formulated using WPI showed a significantly larger particle size in the gastric stage than with SBL (Figure 4). As confirmed in the microscope images, WPI nanoemulsion presented flocculation, while SBL nanoemulsion showed coalescence (Figure 2). Droplets in WPI nanoemulsion became closer due to the WPI proteolysis induced by pepsin during gastric digestion, which may have weakened the viscoelastic interfacial layer [49]. Moreover, the net electrical charge of WPI nanoemulsion was reduced up to 0.55 ± 1.08 mV (Figure 5B) due to the protonation of the amino group (-NH_2_) of WPI at a pH under its pI (5). Such a loss of electric repulsion between the droplets may have favoured the flocculation (Figure 3B). In SBL nanoemulsion, the proximity of the acidic conditions to the p*K*_a_ (≈1.5) of the phospholipid reduced the electrostatic repulsion between the droplets, which may favour the coalescence [50].

WPI nanoemulsions containing sodium alginate at ≥1% concentration presented larger particle sizes in the gastric phase than those without the polysaccharide (Figure 4B). This could be attributed to the formation of WPI-sodium alginate electrostatic complexes due to the attractive forces generated between the negatively charged polysaccharide and the positively charged domains of the protein [51]. SBL nanoemulsions containing sodium alginate showed a less noteworthy particle size increase than the nanoemulsion without the polysaccharide and presented flocculation at all sodium alginate concentrations (Figure 3A and Figure 4A). In these nanoemulsions, since SBL is a high surface-active emulsifier, non-adsorbed polysaccharide molecules may remain in the aqueous phase, promoting depletion flocculation. In this regard, the presence of sodium alginate molecules in the aqueous phase would promote an increase in the attractive forces between droplets due to an osmotic process associated with the exclusion of polysaccharide molecules from a narrow region surrounding the droplets [52,53].

##### Intestinal Phase

At pH 7, WPI and SBL nanoemulsions without sodium alginate exhibited a similar and highly negative electrical charge (Figure 5). On the one hand, the electrical charge of the SBL nanoemulsion was quite similar to that of the gastric phase. On the other hand, WPI nanoemulsion experienced a noteworthy change in the electrical charge from the gastric to the intestine stage because at pH 7, the carboxyl groups (−COO^−^) of WPI became negatively charged, and the amino groups (−NH_2_) became neutral [42]. As previously reported, this promoted the redispersion of droplets that were aggregated in the gastric stage due to electrostatic stabilization [49]. Therefore, in our study, droplets in WPI nanoemulsion may be redispersed when entering the intestinal phase, presenting small particle sizes at early times of intestinal digestion. Conversely, droplets in SBL nanoemulsion could not be redispersed since coalescence is an irreversible phenomenon [32,54]. However, at the end of the intestinal phase, coalescence was also detected in WPI nanoemulsion, as shown in microscope images (Figure 3B). Coalescence occurred during intestinal digestion due to the proteolysis of WPI by pancreatic trypsin, which resulted in small peptides that were unable to stabilize the oil droplets [55].

All WPI nanoemulsions containing sodium alginate presented a larger particle size than those without the polysaccharide at the end of the intestinal phase (Figure 4B). As can be observed in the microscope images, in WPI nanoemulsions with sodium alginate, the coalesced droplets became aggregated in clusters that increased the particle size (Figure 3B). Contrarily, all SBL nanoemulsions containing sodium alginate presented a smaller particle size than those without the polysaccharide (Figure 3A). In polysaccharide-added nanoemulsions, the flocculation rather than coalescence in the gastric phase could have favoured the dispersion of aggregated droplets when entering the intestinal phase. However, as observed in WPI nanoemulsions, during intestinal digestion, aggregation of droplets occurred as a consequence of lipid digestion.

#### 2.2.2. Lipid Digestibility

**Figure 6:**
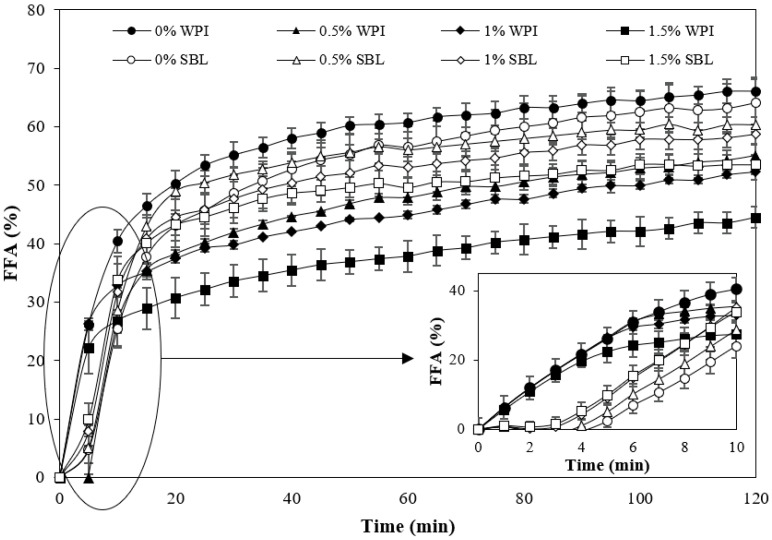
Free fatty acids (FFA) release during the intestinal in vitro digestion of curcumin-loaded nanoemulsions stabilized with lecithin (SBL) or whey protein isolate (WPI) and different sodium alginate concentrations (0, 0.5, 1 and 1.5%).

During the first minutes of intestinal digestion, WPI nanoemulsion showed a faster rate of FFA release than SBL nanoemulsion in the absence of sodium alginate (Figure 6). WPI nanoemulsion may present a higher surface area during the first minutes due to the redispersion of aggregated droplets in the gastric phase (Section 2.2.1). In these nanoemulsions, higher incorporation of lipase molecules at the oil–water interface may occur, bringing lipase into direct contact with the emulsified lipid at early times of lipid digestion [56,57]. Conversely, in SBL nanoemulsions, lipase could not be easily attached to the interface of droplets since SBL present a high surface activity and, in these nanoemulsions, droplets presented a lower surface area, thus showing a slow initial FFA release rate. However, at the end of intestinal digestion, SBL and WPI nanoemulsions without sodium alginate presented the same lipid digestibility, which was about 65%.

**Figure 7:**
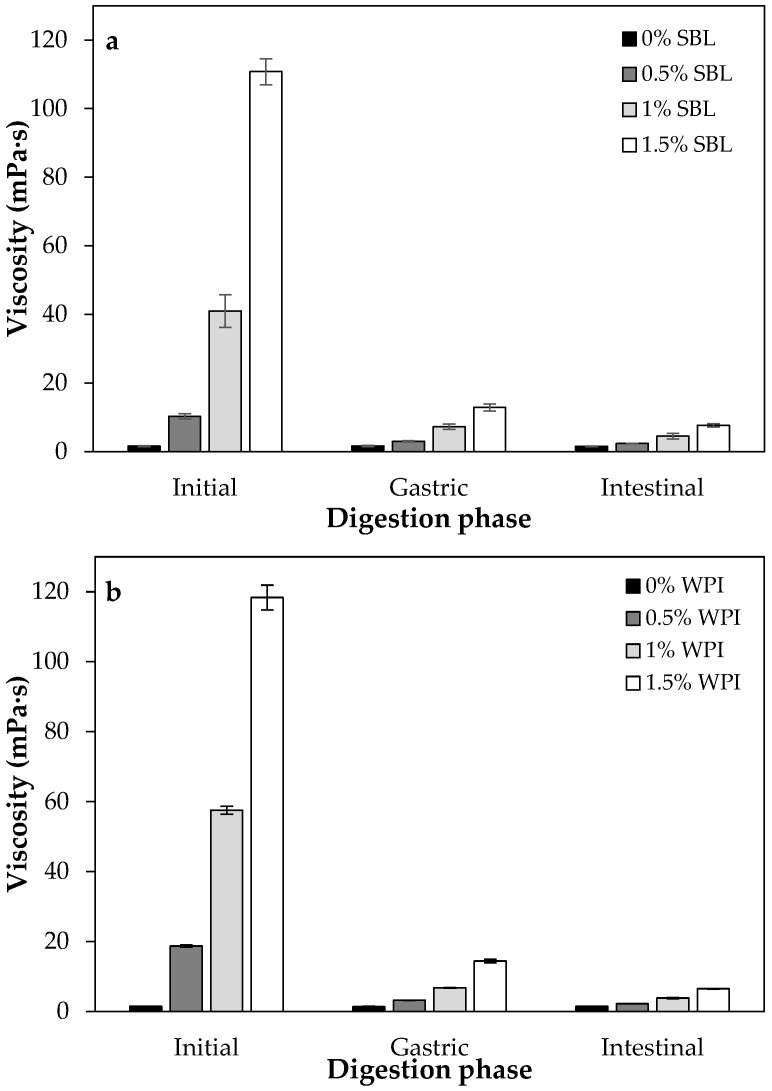
Viscosity of curcumin-loaded nanoemulsions stabilized with (**a**) soybean lecithin (SBL) or (**b**) whey protein isolate (WPI) and different sodium alginate concentrations (0, 0.5, 1 and 1.5%) at different phases of in vitro digestion.

The addition of sodium alginate reduced the lipid digestibility in all nanoemulsions (Figure 6), which is in accordance with previous works [58,59]. The increased viscosity of nanoemulsions containing the polysaccharide (Figure 7) could have slowed down the molecular diffusion, reducing the mobility of the species involved in intestinal digestion [60,61,62]. Moreover, the anionic alginate molecules could strongly bind to cationic calcium ions, which could slow down digestion by preventing the removal of FFA from the surfaces by calcium or by restricting lipase access due to the formation of gels [58]. The initial tendency of WPI and SBL nanoemulsions containing sodium alginate was similar to that observed in nanoemulsions without the polysaccharide. However, at longer times of lipid digestion, the FFA release continued increasing in SBL nanoemulsions with sodium alginate, while it was stabilized in WPI nanoemulsions containing the polysaccharide (Figure 6). At the end of lipid digestion, WPI nanoemulsions containing sodium alginate showed reduced values in comparison to SBL nanoemulsions at the same polysaccharide concentration. As an example, the digestibility of WPI nanoemulsion with 1.5% sodium alginate was 46.4 ± 3.5%, whereas it was 53.6 ± 1.7% in that formulated with SBL at the same polysaccharide concentration. The lower lipid digestibility of WPI nanoemulsions containing sodium alginate could be related to the larger particle size of droplets in the intestinal phase (Figure 1 and Figure 4). As previously mentioned, the coalesced oil droplets in these nanoemulsions became aggregated in clusters of large size. Therefore, these nanoemulsions presented a lower surface area, which reduced the access of lipase to the lipid substrate and, thereby, the lipid digestibility at the end of intestinal digestion [63].

#### 2.2.3. Curcumin Degradation during Digestion

**Figure 8:**
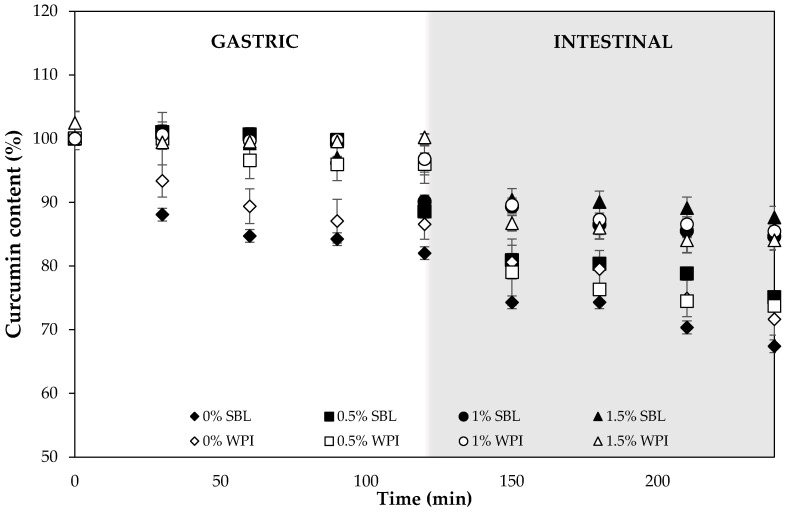
Curcumin content of nanoemulsions stabilized with lecithin (SBL) or whey protein isolate (WPI) and different sodium alginate concentrations (0, 0.5, 1 and 1.5%) during the gastrointestinal in vitro digestion.

During gastric digestion, the degradation of curcumin in nanoemulsions without sodium alginate was lower using WPI (≈13%) than SBL (≈18%) emulsifiers (Figure 8). β-lactoglobulin, which is one of the major components of WPI, has been shown to form complexes with curcumin that protect the compound from degradation during the gastric stage [64]. Peptides resulting from the WPI hydrolysis induced by pepsin are also reported to present antioxidant properties [65,66], which could contribute to reducing curcumin oxidation.

The addition of sodium alginate at the different concentrations used greatly reduced the degradation of curcumin in both SBL and WPI nanoemulsions. Specifically, the lowest degradation (between 0–4%) was observed in WPI nanoemulsion containing 1.5% polysaccharide, which also presented the highest viscosity (Figure 7). The high viscosity proffered by alginate, and the egg-box model gel that this polysaccharide produced due to the presence of Ca^2+^ ions in the gastric fluids [67], may have reduced the diffusion rate of oxidative molecules that can promote curcumin degradation. Moreover, since the pH during the gastric phase was below the p*K*_a_ of sodium alginate (≈3.4), the carboxylic acid groups were non-ionized, thus decreasing the repulsion of the negative charges and causing less polymer chain expansion and low swelling [68]. Therefore, curcumin released from oil droplets may be entrapped in the alginate network, which avoids compound degradation.

The degradation in the intestinal phase was much higher than that observed during the gastric phase (Figure 8). During intestinal digestion, lipid droplets were digested, leading to the loss of the system structure and the release of the encapsulated compound. This resulted in the degradation of curcumin due to the physiological pH of the intestinal fluids [7]. Among nanoemulsions without sodium alginate, WPI nanoemulsions prevented curcumin degradation to a higher extent than that formulated with SBL (Figure 8). The peptides resulting from the WPI hydrolysis during the gastric phase may still be proffering protection to curcumin against degradation due to their antioxidant activity. At the end of the intestinal digestion, WPI nanoemulsion showed a similar curcumin degradation to that formulated with SBL, ≈ 28% and ≈ 33%, respectively. These values are quite similar to those obtained in previous studies with other emulsifiers, such as quillaja saponin [14].

High sodium alginate concentrations continued to prevent curcumin degradation during intestinal digestion (Figure 8). At the end of this stage, the degradation of curcumin was the lowest (15%) in nanoemulsions with high alginate concentrations, while it was up to 32% in nanoemulsions without the polysaccharide. However, it should be noted that curcumin degradation in nanoemulsions containing sodium alginate was higher during intestinal digestion rather than during gastric digestion (Figure 8). Carboxylic acid groups of alginate molecules became ionized at the pH of the intestine, which resulted in a polymer chain expansion and more matrix swelling [68,69]. Therefore, curcumin could be easily released and degraded when exposed to the aqueous media.

#### 2.2.4. Curcumin Bioaccessibility

**Figure 9:**
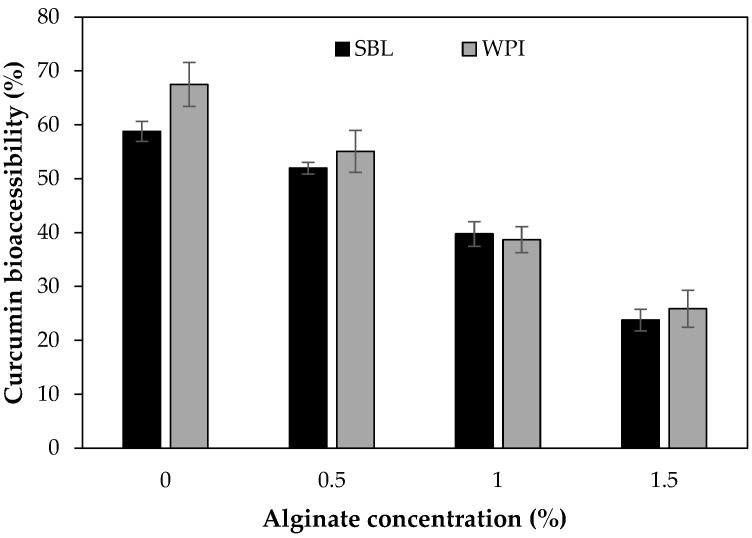
Curcumin bioaccessibility of nanoemulsions stabilized with soybean lecithin (SBL) or whey protein isolate (WPI) and different sodium alginate concentrations (0, 0.5, 1 and 1.5%).

Curcumin bioaccessibility from nanoemulsions without sodium alginate was significantly higher using WPI than with SBL, 67.5 ± 1.8% and 58.8 ± 1.8%, respectively (Figure 9). In our study, the higher bioaccessibility observed in WPI nanoemulsions compared to those with SBL could be attributed to the less curcumin degradation observed in nanoemulsions formulated with the protein emulsifier (Figure 8). Therefore, although both nanoemulsions presented the same lipid digestibility at the end of intestinal digestion (Figure 6), the curcumin content available to be incorporated into the mixed micelles was higher in WPI nanoemulsion than in those with SBL. Previous works reported similar values using whey protein isolate (76%) or lecithin (72%) but also using other emulsifiers such as quillaja saponin (74–79%) [54,70,71]. This indicates that the emulsifier nature has little effect on the bioaccessibility of curcumin.

The presence of sodium alginate noticeably decreased the curcumin bioaccessibility in SBL and WPI nanoemulsions, irrespective of the emulsifier type used. As can be observed in Figure 9, the higher the polysaccharide concentration, the lower the curcumin bioaccessibility. In fact, the bioaccessibility was reduced by 15%, 35% and 60% by adding 0.5%, 1% and 1.5% sodium alginate, respectively. The reduced curcumin bioaccessibility that presented sodium alginate nanoemulsions in comparison to non-added sodium alginate nanoemulsions can be related to the digestibility results. In polysaccharide-added nanoemulsions, the reduced digestibility promoted the presence of more undigested oil at the end of the intestinal digestion that can be entrapping curcumin [72,73].

At the same sodium alginate concentration, SBL and WPI nanoemulsions showed the same curcumin bioaccessibility (Figure 9), although the digestibility of WPI nanoemulsions was lower (Figure 6). Based on the findings of previous works, it could be expected that WPI nanoemulsions presented reduced bioaccessibility due to their low digestibility. However, as mentioned in the previous Section 2.2.3, WPI nanoemulsions were more effective in preventing curcumin degradation during the gastric stage (Figure 8). Therefore, WPI nanoemulsions containing sodium alginate presented the highest curcumin concentration during intestinal digestion. As a result, although polysaccharide nanoemulsions containing WPI as an emulsifier presented less lipid digestibility than those with SBL, both emulsifier-type nanoemulsions presented the same curcumin bioaccessibility.

## 3. Materials and Methods

### 3.1. Materials

Curcumin, pepsin (from porcine gastric mucosa), pancreatin (from porcine pancreas), and bile extract (porcine) were obtained from Sigma-Aldrich, INC (St. Louis, MO, USA). Corn oil (Koipesol Asua, Deoleo, Spain) was purchased from a local supermarket. Soybean lecithin (SBL) was acquired from Alfa Aesar (Thermo Fisher Scientific, Waltham, WA, USA). Whey protein isolate (WPI) was kindly provided by El Pastoret de la Segarra, S.L. (Spain). Sodium alginate (MANUCOL^®^ DH) was provided by FMC Biopolymers Ltd. (Scotland, UK). Ultrapure water obtained from a Milli-Q filtration system was used to prepare all solutions.

### 3.2. Methods

#### 3.2.1. Nanoemulsion Preparation

To obtain the lipid phase, curcumin was solubilized in corn oil (1 mg·g^−1^) by stirring for 20 min at 60 °C and sonicating for 20 min. To formulate the aqueous phase, sodium alginate (0, 0.5, 1 or 1.5 % *w*/*w*) was added into ultrapure water and stirred overnight at room temperature (≈20 °C). Then, SBL or WPI was added (5% *w*/*w*), and the mixture was stirred. To formulate the coarse emulsion, the lipid phase (5% *w*/*w*) and the aqueous phase (95% *w*/*w*) were mixed using an Ultra-Turrax at 11,000 rpm for 2 min (Janke & Kundel, Staufen, Germany). Finally, to obtain the nanoemulsion, the coarse emulsion was treated with a UP400S sonifier (Hielscher Ultrasound Technology, Teltow, Germany) of 400 W nominal power and a frequency of 24 kHz equipped with a 22 mm sonotrode for 3 min at 100% of amplitude.

#### 3.2.2. Physicochemical Characterization

The particle size and particle size distribution of nanoemulsions were measured using a Mastersizer 3000 (Malvern Instruments Ltd., Worcestershire, UK). Samples were diluted in ultrapure water and stirred in the dispersion unit with a constant speed of 1800 rpm. The mean particle size was expressed as surface area mean diameter (d_32_) in micrometers (µm), fixing a refractive index of the corn oil of 1.473 and 1.333 for water.

The ζ-potential was measured by phase analysis light scattering (PALS) using a Zetasizer laser diffractometer (NanoZS Malvern Instruments Ltd. Worcestershire, UK). Prior to the analysis, nanoemulsions were diluted (1:100) in ultrapure water and placed in a capillary cell equipped with two electrodes to assess the electrophoretic mobility of the particles. The results were reported in millivolts (mV).

To study the encapsulation efficiency (EE), 10 mL of nanoemulsion was placed inside a dialysis tubing cellulose membrane of 43 mm × 27 mm (Sigma-Aldrich, Darmstadt, Germany). Then, the membrane was inserted into a centrifuge tube containing 20 mL of ethanol and centrifuged at 2000 rpm for 10 min. Finally, the non-encapsulated curcumin content was quantified using a UV-visible spectrophotometer (CECIL CE 2021; Cecil Instruments Ltd., Cambridge, UK) at 425 nm. The encapsulation efficiency (%) was calculated using Equation (1) [74]:

(1) $$\mathrm{EE}\;(\%)=\frac{\mathrm{Total}\;\mathrm{amount}\;\mathrm{of}\;\mathrm{curcumin}\;-\;\mathrm{Free}\;\mathrm{curcumin}}{\mathrm{Total}\;\mathrm{amount}\;\mathrm{of}\;\mathrm{curcumin}}\;\times \;100$$

where the total amount of curcumin is the initial concentration added in nanoemulsions, and the free curcumin is the concentration of the compound that was not loaded in nanoemulsions.

The apparent viscosity of nanoemulsions was determined using an SV-10 vibro-viscometer (A&D Company, Tokyo, Japan), which produces a vibration of 30 Hz and a constant amplitude of 0.4 mm at controlled room temperature. The results were expressed in mPa·s.

#### 3.2.3. Stability of Nanoemulsions

Stability of emulsions was studied using an optical scan analyzer Turbiscan MA 2000 (Formulaction, Toulouse, France), which can measure the static stability of samples without destruction and detect the cause of instability (flocculation, coalescence, sedimentation or creaming) by the multiple light scattering technique. A sample of 7 mL was introduced into a glass cylindrical cell and analyzed by a light beam emitted in near-infrared wavelength, which scanned vertically from the bottom to the top of the sample cell. Two synchronous optical sensors receive light backscattered by the sample (45° from the incident radiation). In this study, the variation of backscattering (BS) during 21 days at 4 °C was studied to assess the stability of the nanoemulsions over time.

#### 3.2.4. Curcuminoid Extraction from Nanoemulsions and Quantification

To extract curcumin from nanoemulsions, a previously reported method with some modifications was used [11]. First, aliquots of 250 µL of the sample were mixed with 1000 µL of ethyl acetate and vortexed for 1 min and centrifuged at 9000 rpm for 10 min at 4 °C. Afterwards, the upper organic layer was collected and evaporated under N_2_ and stored at −40 °C. The quantification of curcumin was carried out using an HPLC system equipped with a 600 Controller and a diode array detector (Waters, Milford, MA) following a previously described method with some modifications [75]. Before injecting the samples into the HPLC, they were reconstituted with 1 mL of methanol and filtered. Curcumin was identified using a reverse-phase C18 Spherisorb^®^ ODS2 (5 µm) stainless steel column (4.6 mm × 250 mm) at room temperature. The separation was performed with a linear gradient elution 2% aqueous acetic acid (solvent A) and acetonitrile (solvent B). Detection was by UV spectroscopy at a wavelength of 425 nm, and the flow rate was 1 mL/min. UV-Vis spectral data and their retention times were used to determine the curcumin present in the vials being quantified by comparing them with an external curcumin standard.

#### 3.2.5. In Vitro Digestion

To simulate the human digestion process, an in vitro gastrointestinal digestion based on an international consensus method [76] was used. 

The protocol included both gastric and small intestinal phases. The mouth phase was not performed since nanoemulsions were liquid. To perform the gastric phase, 20 mL of nanoemulsion was mixed 1:1 with simulated gastric fluids containing pepsin (2000 U/mL) and 10 μL of a CaCl_2_ solution (0.3 M). Then, the pH was adjusted to 3 using HCl (1 M), and the mixture was placed into an incubator at 37 °C for 2 h while shaking at 100 rpm. To simulate the intestinal phase, a pH-stat device was used. Once the gastric phase was completed, the gastric sample was placed in a 37 °C water bath and mixed 1:1 with simulated intestinal fluids containing 10 mM bile solution. Then, the pH was adjusted to 7 with NaOH (1 M), and pancreatin and lipase enzymes were added to the final mixture to achieve 100 U/mL of trypsin and 2000 U/mL pancreatic lipase. The pH of the sample was maintained at 7 by adding NaOH (0.25 M) constantly for 2 h. According to previous authors [77], the FFA (%) was determined using Equation (2):

(2) $$\mathrm{FFA}\;\left(\%\right)=\frac{V_{\mathrm{NaOH}}{\times \;C}_{\mathrm{NaOH}}{\;\times \;M}_{\mathrm{oil}}}{{2\;\times \;m}_{\mathrm{oil}}}\;\times 100$$

where V_NaOH_ is NaOH volume (L) used during the intestinal digestion, C_NaOH_ is NaOH molarity (0.25 mol/L), M_oil_ is corn oil molecular weight (800 g/mol), and m_oil_ is corn oil total weight present in the emulsions (g).

#### 3.2.6. Curcumin Degradation

To study the degradation of curcumin along the simulated gastrointestinal tract, 1 mL of digesta was collected at specific digestion times (0, 30, 60, 90, 120, 150, 180, 210 and 240 min). Then curcumin was extracted and quantified as described in Section 3.2.4 to obtain curcumin content values. Finally, the curcumin content in the aliquots collected at different digestion times was obtained according to Equation (3):

(3) $$\mathrm{Curcumin}\;\mathrm{content}\;\left(\%\right)=\frac{C_{\mathrm{digesta}}}{C_{\mathrm{initial}}}\;\times 100$$

where C_digesta_ is the curcumin concentration (mg/mL) in the digesta and C_initial_ is the initial curcumin concentration (mg/mL) in the nanoemulsion.

#### 3.2.7. Bioaccessibility

To study the curcumin bioaccessibility, after the digestion process, the digested nanoemulsions were centrifuged at 9000 rpm for 30 min at 4 °C to obtain the micellar fraction. Then, an extraction and quantification of the curcumin content was performed both in the initial and micellar fraction following the method described in Section 3.2.4. Finally, curcumin bioaccessibility was calculated according to Equation (4):

(4) $$\mathrm{Bioaccessibility}\left(\%\right)=\frac{C_{\mathrm{micelle}}}{C_{\mathrm{initial}}}\;\times 100$$

where C_micelle_ is the curcumin concentration (mg/mL) in the micellar fraction and C_initial_ is the initial curcumin concentration (mg/mL) in the nanoemulsion.

#### 3.2.8. Optical Microscopy

Images of curcumin-loaded nanoemulsions were obtained using an optical microscope (Olympus BX41, Olympus America Inc., Melville, NY, USA) with a 100× objective lens. The images were obtained using a digital camera (Olympus DP74) and processed with the software CellSens (Olympus).

#### 3.2.9. Statistical Analysis

All experiments were assayed in duplicate, and three repetitions of each analysis were carried out on each parameter in order to obtain mean values. Analysis of the variance (ANOVA) was performed to compare treatments. Least significant difference (LSD) test was employed to determine differences between means. The confidence interval was set at 0.95, and all results were analyzed using the Statgraphics Plus v.5.1 Windows package (Statistical Graphics Co., Rockville, MD, USA).

## 4. Conclusions

This study has shown that both lipidic and protein emulsifiers can be used to produce stable nanoemulsions with a high curcumin encapsulation efficiency (≈96%). The addition of sodium alginate influenced the physical properties of nanoemulsions and increased their stability over time at concentrations of 1–1.5%. During gastrointestinal digestion, WPI better-prevented curcumin degradation than SBL, which resulted in a higher curcumin bioaccessibility. The addition of sodium alginate greatly reduced curcumin degradation during digestion, especially when used with the protein emulsifier during the gastric phase as a consequence of WPI-polysaccharide complex formation. However, the presence of the polysaccharide reduced the digestibility of nanoemulsions and, thereby, curcumin bioaccessibility. Sodium alginate seems to be a promising stabilizer for curcumin nanoemulsions when used at 1–1.5%, greatly preventing curcumin degradation during gastrointestinal digestion. However, a 1% concentration seems to be the most suitable, as it reduces curcumin bioaccessibility to a lesser extent.

## Figures and Tables

**Table 1:** Initial characterization of soybean lecithin and whey protein isolate curcumin-loaded nanoemulsions containing different sodium alginate concentrations.

Emulsifier Type	Alginate (%)	*d_32_* (µm)	ζ-Potential (mV)	Encapsulation Efficiency (%)	Viscosity (mPa·s)
Soybean lecithin	0	0.344 ± 0.01 c	−47.1 ± 3.3 c	97.0 ± 0.8 ab	1.59 ± 0.13 a
	0.5	0.308 ± 0.02 b	−65.7 ± 2.6 b	97.1 ± 1.0 ab	10.28 ± 0.79 b
	1	0.288 ± 0.02 a	−76.6 ± 3.6 a	97.5 ± 0.2 b	41.01 ± 4.76 c
	1.5	0.288 ± 0.02 a	−77.8 ± 4.3 a	96.7 ± 0.3 a	110.78 ± 3.79 d
Whey protein isolate	0	0.487 ± 0.01 a	−37.3 ± 2.5 d	95.1 ± 0.2 a	1.44 ± 0.03 a
	0.5	0.499 ± 0.03 b	−43.9 ± 2.3 c	95.2 ± 0.7 a	18.72 ± 0.30 b
	1	2.080 ± 0.11 c	−52.1 ± 1.1 b	95.6 ± 1.1 a	57.53 ± 1.16 c
	1.5	2.251 ± 0.27 d	−57.7 ± 1.4 a	95.7 ± 0.2 a	118.33 ± 3.56 d

Values are expressed as mean ± standard deviation. Different letters indicate significant differences (*p* < 0.05) between nanoemulsions with different sodium alginate concentrations.

## Data Availability

The data used to support the findings of this study can be made available by the corresponding author upon request.
